# Outcomes of Adjuvant Radiation Therapy in Penile Cancer

**DOI:** 10.1016/j.adro.2025.101986

**Published:** 2025-12-25

**Authors:** Gerim Prasai, David K. Simson, Sauharda Lohani, Sarthak Tandon, Varghese Antony, Parveen Ahlawat, Shaifali Mahajan, Anurag Mehta, Vineet Talwar, Ashish Khanna, Amitabh Singh, Munish Gairola, Sudhir Rawal, Jaskaran Singh Sethi

**Affiliations:** a Department of Radiation Oncology, Kathmandu Cancer Center, Kathmandu, Nepal; b Department of Radiation Oncology, Rajiv Gandhi Cancer Institute and Research Center, New Delhi, India; c Department of Radiation Oncology, Nepal Cancer Hospital and Research Center, Kathmandu, Nepal; d Department of Pathology, Rajiv Gandhi Cancer Institute and Research center, New Delhi, India; e Department of Medical Oncology, Rajiv Gandhi Cancer Institute and Research center, New Delhi, India; f Department of GenitoUro-Oncology, Rajiv Gandhi Cancer Institute and Research center, New Delhi, India

## Abstract

**Purpose:**

This study evaluates the long-term outcomes of adjuvant radiation therapy (RT) in patients with penile cancer treated over 14 years.

**Methods and Materials:**

In this retrospective cohort study, patients with squamous cell carcinoma of the penis who underwent surgery followed by adjuvant RT with or without concurrent chemotherapy from January 2010 to December 2022 were included, with follow-up continuing until December 2023. Primary endpoints were overall survival (OS) and recurrence-free survival (RFS), with additional analyses of toxicity and recurrence patterns. Survival rates were estimated using the Kaplan-Meier method, and univariate analysis was performed using Cox proportional hazards regression to assess the associations between clinical variables and outcomes (OS and RFS). Hazard ratios with 95% CIs were calculated, and a *P* value of <.05 was considered statistically significant.

**Results:**

A total of 30 patients met the selection criteria. The median age was 57 years (IQR, 52-63), with a median follow-up of 84 months (95% CI, 74.02-92.81 months). The median OS was 77.2 months, while the median RFS was not reached. Five-year and 7-year survival rates were 52% and 46%, respectively. The 5-year RFS was 66%. Among 8 patients with recurrence, 6 had locoregional and 2 had distant metastasis. RT was generally well tolerated, with lymphedema as the most common side effect.

**Conclusions:**

Despite adjuvant RT, survival outcomes remain suboptimal, with high rates of locoregional relapse. These findings underscore the need for improved treatment strategies.

## Introduction

Penile cancer is a rare malignancy with an incidence of ≤1/100,000 population, accounting for 0.5% of cancers in India.^1^ The cornerstone of treatment is surgery, often followed by adjuvant therapies when indicated.^2^ Adjuvant treatment options include radiation therapy (RT), with or without concurrent chemotherapy, while some studies suggest neoadjuvant chemotherapy (NACT) for locally advanced cases, particularly when nodal size exceeds 4 cm or nodes are fixed.^3^ Among adjuvant therapies, RT has shown promising results in improving locoregional control.^4^

However, its optimal integration into treatment remains unclear due to limited evidence, largely stemming from the rarity and heterogeneity of the disease. Owing to the rarity of penile cancer, conducting adequately powered prospective or randomized trials remains challenging, and most available evidence is derived from retrospective studies with small patient cohorts.

This study aims to address these gaps by presenting 14 years of experience with adjuvant RT for locally advanced penile cancer, focusing on survival outcomes, recurrence patterns, and treatment-related toxicity.

## Methods and Materials

### Study design and setting

This retrospective cohort study was conducted at a high-volume tertiary cancer center, adhering to the principles of the Declaration of Helsinki. The study period spanned from January 2010 to December 2023, with patient identification from January 2010 to December 2022. Follow-up continued until December 2023.

### Timeline definitions

The pre-index period encompassed the time from patient registration to the assignment of adjuvant therapy. The index date marked the assignment of therapy by the multidisciplinary tumor board (MDT). Finally, the postindex period extended from the index date to the data cut-off date or death.

### Participants and data sources

**Figure 1:**
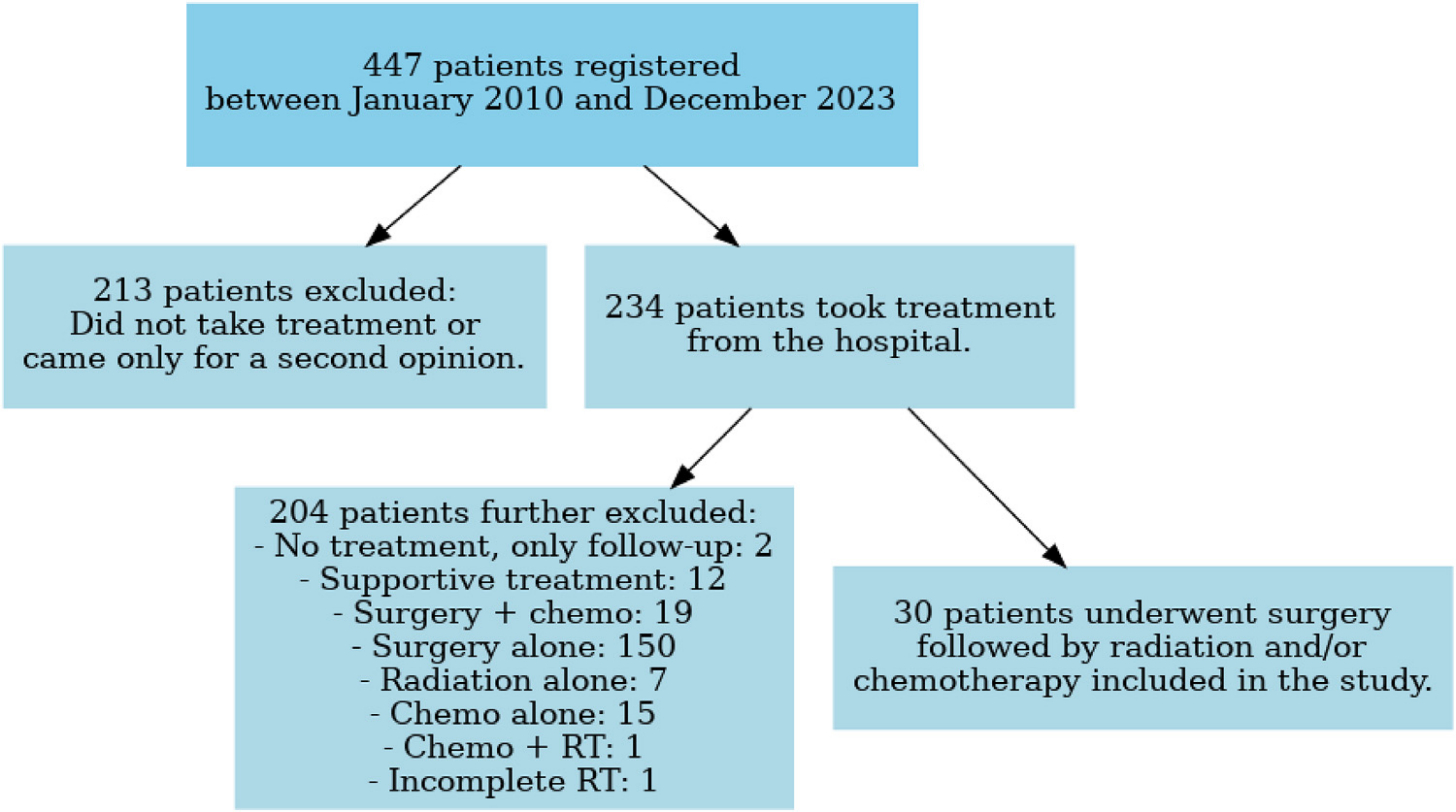
Flow diagram illustrating the attrition process during the study period. *Abbreviation*: RT = radiation therapy.

Patients with histology-proven squamous cell carcinoma of the penis who underwent surgery followed by adjuvant RT, with or without concurrent chemotherapy, were included. Data were retrieved from the institutional electronic database using International Classification of Diseases-10 code C60 and treatment codes RG501 to RG514.^5^ Patient details, including demographic, clinical, staging, treatment, and outcome data, were collected from electronic medical records. Of note, during the study period, 150 patients underwent surgery alone. Of these, 9 patients who met the criteria for adjuvant RT but were not included in the study cohort: 2 received RT elsewhere, 3 declined treatment, and 4 progressed with prepubic/inguinal skin nodules while awaiting RT. The participant attrition process is depicted in Fig. 1.

### Selection criteria

Inclusion criteria required patients to be at least 18 years old, have an Eastern Cooperative Oncology Group performance status of ≤2, and have completed inguinal and/or pelvic nodal dissection with adjuvant RT with at least 1 year of follow-up data. Patients diagnosed outside the hospital were included after review of the pathology slides and tissue blocks. The intent of treatment for all patients was curative, and all cases were discussed in the MDT meeting.

### Follow-up

During RT, patients attended weekly follow-up appointments, and acute toxicities were documented. Following completion of RT, patients are reviewed every 3 months for the first 2 years, every 6 months until the fifth year, and annually thereafter. Since 2019, our institutional protocol includes positron emission tomography contrast-enhanced computed tomography (PET-CECT) of the pelvis at 3 months posttreatment, followed by PET-CECT every 6 months for the first 5 years and annually thereafter. During interim visits in the first 2 years, ultrasound of the whole abdomen, scrotum, genitalia, and inguinal regions, together with a chest x-ray, is routinely performed. Before 2019, instead of PET-CECT, patients underwent CECT of the chest, abdomen, and pelvis, with magnetic resonance imaging (MRI) performed selectively when clinically indicated. At each follow-up, a detailed physical examination is also performed, with particular attention to detecting cutaneous metastases that may not be evident on imaging. Data on late toxicities and survival were recorded from the clinic visits or telephone follow-ups.

### Treatment protocols

#### Surgery

MRI was performed for local staging in all patients before surgery. For metastatic workup, CECT of the thorax, abdomen, and pelvis was routinely performed. Since 2019, PET/CT has been included in the workup for all patients with cN2 or higher stage during workup. No surgical staging of the inguinal basins, including dynamic sentinel node sampling, was performed preoperatively. Surgical procedures included partial or total penectomy, depending on the extent of the disease, and inguinal lymph node dissection (ILND) performed using minimally invasive (robotic-assisted/video-assisted) or open techniques. Pelvic lymph node dissection (PLND) was performed in cases with clinically or pathologically positive inguinal nodes, as clinically indicated.

#### MDT discussion

In all node-positive patients, inguinal nodes were treated with adjuvant RT. The decision to include pelvic nodes was based on multiple factors, such as the presence of multiple involved nodes, pathologic risk factors, the number of lymph nodes dissected, and the patient's performance status. The decision to administer concurrent chemotherapy was made, taking into account the patient's clinical and pathologic features, including comorbidities.

#### Radiation therapy

The institutional protocol for RT included a dose of 45 to 50.4 Gy to the inguinal and/or pelvic nodal region, with an additional boost of 54 Gy in the presence of extra nodal extension (ENE) or multiple high-risk pathologic features; however, further RT boost dose was considered in selected cases as per the treating clinical radiation oncologist team and after MDT discussion. The standard radiation fields included the regional lymphatic basins (inguinal and/or pelvic) based on the clinical stage and nodal status.^4^ The decision to treat common iliac nodes was taken based on the individual patient’s clinicopathological features. The penile stump was included in the treatment field if the surgical margin was positive. Until 2022, the fields encompassed the inguinal and/or pelvic regions as per institutional protocol. From 2023 onward, the prepubic fat space was also included in the clinical target volume, following observed patterns of recurrence in this area.^4^ Techniques used included 2-dimensional (2D) conventional RT, 3-dimensional conformal RT (3DCRT), intensity modulated RT, and image guided RT, with the choice of technique dependent on the clinical scenario and technological availability. The details of the techniques and setup verifications performed using cone beam CT or electronic portal imaging device are provided in Table 1.

**Table 1:** Demographic and tumor characteristics of the study population

Characteristic	Value (N = 30), n (%)
Age (y), median (IQR)	57.0 (52.0-63.0)
pT size (cm), median (IQR)	4.0 (2.45-4.50)
pN size (cm), median (IQR)	2.0 (1.70-3.13)
pT stage	
T1	5 (16.7%)
T2	11 (36.7%)
T3	13 (43.3%)
T4	1 (3.3%)
pN stage	
N0	2 (6.7%)
N1	1 (3.3%)
N2	4 (13.3%)
N3	23 (76.7%)
cN stage	
cN1	8 (26.6%)
cN2	8 (26.6%)
cN3	14 (46.7%)
Stage (AJCC 8th edition)	
IIA	1 (3.3%)
IIB	1 (3.3%)
IIIA	1 (3.3%)
IIIB	4 (13.3%)
IV	23 (76.7%)
Grade	
Low-grade (grade I-II)	24 (80.0%)
High-grade (grade III)	6 (20.0%)
PNI	
Yes	12 (40.0%)
No	18 (60.0%)
LVI	
Yes	16 (53.3%)
No	14 (46.7%)
ENE	
Yes	23 (76.7%)
No	7 (23.3%)
Type of penectomy	
Partial penectomy	19 (63.3%)
Total penectomy	11 (36.7%)
Lymph node dissection	
Open	12 (40.0%)
Robotic/video-assisted	18 (60.0%)
Number of lymph nodes dissected, median (IQR)	41 (30-52)
Intent of chemotherapy	
NACT	1 (3.3%)
Concurrent	7 (23.3%)
Adjuvant	1 (3.3%)
Setup verification and technique	
Daily CBCT for IGRT	21 (70%)
Twice a week CBCT for IMRT	6 (20%)
EPID for 2D and 3D-CRT	3 (10%)

*Abbreviations*: 2D and 3D-CRT = 2-dimensional and 3-dimensional conformal radiation therapy; AJCC = American Joint Committee on Cancer; CBCT = cone beam computed tomography; cN = clinical nodal; ENE = extranodal extension; EPID = electronic portal imaging device; IGRT = image guided radiation therapy; IMRT = intensity modulated radiation therapy; LVI = lymphovascular invasion; NACT = neoadjuvant chemotherapy; pN = pathological nodal; PNI = perineural invasion; pT = pathological tumor.

#### Chemotherapy

Concurrent regimens typically included cisplatin-based therapies (40 mg/m^2^ weekly), either alone or in combination, for high-risk cases.

Those patients who did not undergo NACT and underwent surgery with pathologic nodal positivity were considered for adjuvant chemotherapy as per MDT decision. Regimens used were TIP (paclitaxel, ifosfamide, cisplatin) or 5-FU (5 Flurouracil) and cisplatin-based chemotherapy.

### Measurable variables

Variables like age, tumor size and nodal size were treated as continuous, while others such as type of surgery (total vs partial penectomy), tumor stage (T stage), nodal stage (N stage), tumor grade (low-grade [grades I-II] vs high-grade [grade III]), and presence of adverse pathologic features like perineural invasion (PNI), lymphovascular invasion (LVI), or ENE were treated as categorical variables.

### Exposure of interest

The primary exposure of interest was the administration of adjuvant RT.

### Endpoints and analysis

The primary endpoints were overall survival (OS) and recurrence-free survival (RFS). OS was defined as the time from diagnosis to death or date of last follow-up, and RFS as the time from diagnosis to recurrence. Recurrences were classified as locoregional or distant. Toxicities were graded using National Cancer Institute Common Terminology Criteria for Adverse Events v5.0, with acute toxicities defined as those occurring within 3 months of RT and late toxicities beyond 3 months.^6^, ^7^, ^8^

### Statistical methods

Descriptive statistics are summarized as continuous (mean/median) and categorical (frequencies/percentages) variables. The median follow-up was calculated using the inverse Kaplan-Meier method. Kaplan-Meier analysis was used to estimate survival, and univariate Cox regression was used to assess associations between variables and outcomes. Survival curves were truncated once the number of patients at risk fell below 10, consistent with established recommendations to avoid unstable estimates in the tail of the distribution. A multivariate analysis was consciously not performed due to the low event-to-variable ratio in our data set. Hazard ratios (HRs) with 95% CIs were calculated; *P* < .05 was considered statistically significant.

All statistical analyses, except survival curve plotting, were performed using IBM SPSS Statistics (version 25; IBM Corp.). Survival curves for OS and RFS were generated using R (R Foundation for Statistical Computing) with the RStudio interface.

### Ethical considerations

Our study received a waiver from the ethical board as it is a retrospective analysis. No special consent was obtained for this study; however, standard treatment-specific consents (RT, chemotherapy, or surgery) were obtained in accordance with institutional norms and council requirements. There were no financial disclosures or external funding associated with this study.

### Bias and confounding

Potential sources of bias included patient demographics, treatment compliance, and variations in management. Efforts were made to minimize bias by recording confounders such as age, comorbidities, and Eastern Cooperative Oncology Group performance status. The study adhered to Strengthening the Reporting of Observational Studies in Epidemiology guidelines to ensure transparent and comprehensive reporting.

### Sensitivity analyses

Sensitivity analyses were not performed due to the small sample size and retrospective nature of the study. This limitation may have impacted the robustness of the findings, as certain unmeasured variables or interactions could not be thoroughly explored.

## Results

A total of 30 men who met the selection criteria were included in the study. The median age was 57 years (IQR, 52-63), with 63.3% undergoing partial penectomy and 36.7% undergoing total penectomy. All patients were HIV negative. Human papillomavirus (HPV) testing was introduced since 2018, after which 9 patients underwent testing. Of these, 2 (2/9) were positive for HPV. Testing was performed using immunohistochemistry for p16.

Eighty percent had low-grade tumors (grades 1 and 2), and 20% had high-grade (grade 3) tumors. Most patients in the cohort presented with pT3 disease (43.3%) and pN3 disease (76.7%). The complete list of cN, pT, and pN staging, along with other demographic and tumor characteristics of the study population, is given in Table 1. Reliable cT staging information was not available and therefore not included in this study. The median primary tumor size was 4.0 cm (IQR, 2.45-4.50 cm), and the median nodal size was 2.0 cm (IQR, 1.70-3.13 cm).

Inguinal lymph node positivity was observed in 93.3% of patients, with 70% having involvement of 2 or more nodes. A total of 23 patients were classified as pN3. All 23 patients had ENE, and 4 of these also demonstrated pelvic nodal metastasis, with ENE present in inguinal and/or pelvic nodes. This is further discussed below.

In our cohort, 2 patients were pathologically N0 yet received adjuvant RT. One patient had an R1 resection at the primary site, for which adjuvant RT to the primary operative bed was administered. The second patient had only 5 lymph nodes dissected, raising concern for potential understaging; following an MDT board review, adjuvant RT was offered in this context.

The median radiation dose administered was 50.4 Gy (minimum 30.6 Gy, maximum 65 Gy). One patient discontinued RT at 30.6 Gy due to poor tolerance. The median interval between surgery and adjuvant RT was 60 days (minimum 40 days, maximum 284 days). Most delays were related to postoperative wound-healing. In the patient with the longest interval (284 days), RT was delayed due to the completion of 6 cycles of adjuvant chemotherapy before RT. Concurrent chemotherapy was administered to 26.6% of patients, predominantly cisplatin-based regimens, while 3.3% received NACT (Table 1).

The median follow-up duration for the cohort was 84 months (95% CI, 74.02-92.81 months). The median OS was 77.2 months, with 5-year and 7-year survival rates of 52% and 46%, respectively. The 5-year RFS is 66%. Median RFS was not reached.

**Figure 2:**
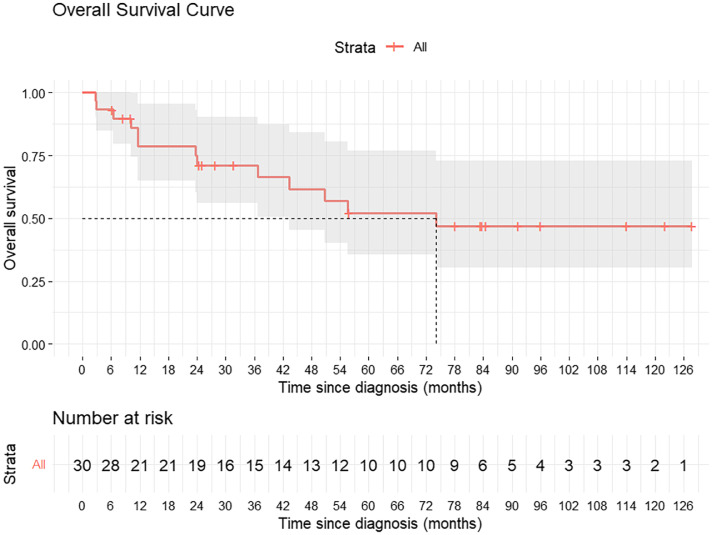
Kaplan-Meier curve for overall survival (OS).

**Figure 3:**
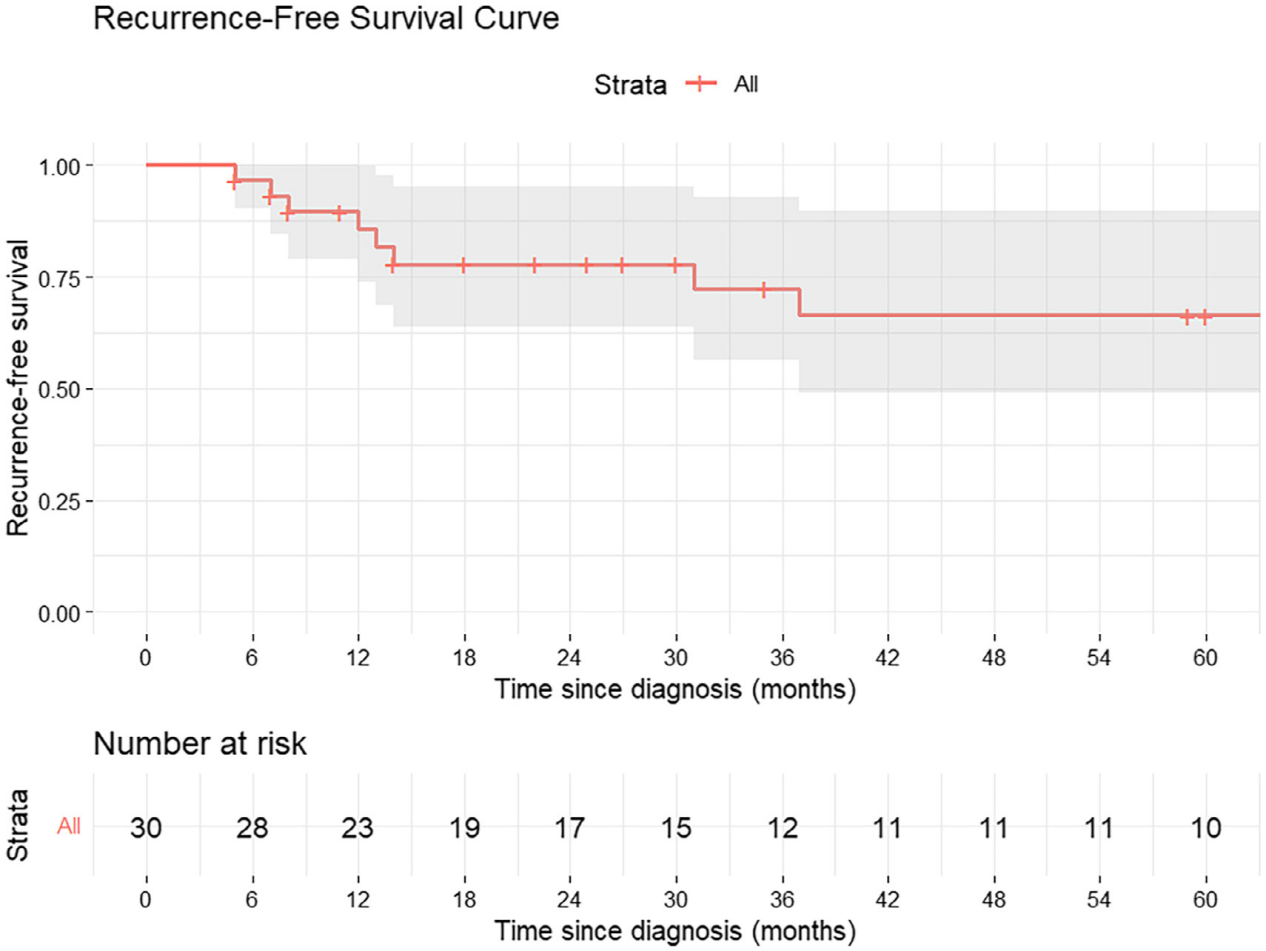
Kaplan-Meier curve showing recurrence-free survival (RFS).

During follow-up, 8 of 30 patients (26.6%) developed recurrence. Six patients (20%) experienced locoregional recurrence, while 2 patients (6.6%) developed distant metastasis. Among the locoregional failures, 2 patients (33.3%) had recurrence in the prepubic fat region (2/6). Overall, this accounted for 25% of all recurrences (2/8). Two patients developed distant metastases in the lungs (Table 2). Figure 2 illustrates the Kaplan-Meier curve for OS, while Fig. 3 depicts RFS.

**Table 2:** Recurrence pattern observed in the study

Patient number	Locoregional recurrence	Distant
Serial no 1	-	Lung
Serial no 2	Penile stump	-
Serial no 3	Penile stump + inguinal node	-
Serial no 9	-	Lung
Serial no 11	Pubic soft tissue	-
Serial no 12	Pubic soft tissue	-
Serial no 22	Penile stump + inguinal node	-
Serial no 25	Penile stump + inguinal node	-

**Table 3:** Univariate analysis for recurrence-free survival (RFS)

Variables	Subgroup	Hazard ratio (CI)	Significance (*P* value)
pT stage	T1		
	T2	1.358 (0.231-7.979)	.735
	T3	0.854 ((0.155-4.692)	.856
	T4	4.285 (0.350-52.495)	.255
pN stage	N0		
	N1	1.67 (0.46-3.56)	.189
	N2	1.58 (0.34-1.2)	.795
	N3	2.3 (0.67-5.89)	.899
PNI	No Yes	0.893 (0.100-7.965)	.919
LVI	No Yes	0.328 (0.041-2.611)	.292
Grade	Low (grades I and II) High (grade III)	1.065 (0.093-12.246)	.960
Penectomy	Total partial	1.492 (0.254-8.774)	.658
ENE	No Yes	3.382 (0.040-285.078)	.590
T size	Continuous variable	1.297 (0.979-1.717)	.070
N size	Continuous variable	0.953 (0.748-1.214)	.697

*Abbreviations*: ENE = extranodal extension; LVI = lymphovascular invasion; N = nodal; pN = pathological nodal; PNI = perineural invasion; pT = pathological tumor; T = tumor.

**Table 4:** Univariate analysis for overall survival (OS)

Variables	Subgroup	Hazard ratio (CI)	Significance (*P* value)
pT stage	T1		
	T2	1.858 (0.344-10.026)	.471
	T3	0.954 (0.183-4.886)	.947
	T4	6.271 (0.497-79.148)	.156
pN stage	N0		
	N1	1.12 (0.38-1.78)	.899
	N2	1.39 (0.74-2.98)	.455
	N3	1.22 (0.45-3.89)	.789
PNI	No Yes	0.483 (0.082-2.834)	.420
LVI	No Yes	0.403 (0.079-2.056)	.274
Grade	Low (grades I and II) High (grade III)	2.133 (0.283-16.105)	.463
Penectomy	Total partial	0.611 (0.121-3.096)	.552
ENE	No Yes	0.752 (0.028-20.480)	.866
T size	Continuous variable	1.290 (0.997-1.66)	.053
N size	Continuous variable	0.998 (0.821-1.212)	.981

*Abbreviations*: ENE = extranodal extension; LVI = lymphovascular invasion; N = nodal; pN = pathological nodal; PNI = perineural invasion; pT = pathological tumor; T = tumor.

On univariate analysis (Table 3, Table 4), none of the clinicopathologic variables reached statistical significance for RFS or OS, although several demonstrated trends consistent with known prognostic patterns. Tumor size, analyzed as a continuous variable, showed a trend toward worse outcomes, approaching significance for OS (HR 1.290; 95% CI, 0.997-1.660; *P* = .053; Table 4) and for RFS (HR 1.297; 95% CI, 0.979-1.717; *P* = .070; Table 3). Nodal size did not show a significant association with either RFS or OS. Other pathologic features, including T stage, N stage, grade, LVI, PNI, and ENE, did not reach statistical significance. Acute toxicities were evaluated, with grades 1-2 skin reactions observed in 83.4% of patients and grade 3 reactions in 16.6%. Lymphedema (grade 1) occurred in 33% of patients, and exacerbation of hemorrhoids was noted in 6.7%. Hematological toxicities, including anemia and neutropenia, were more common among patients receiving concurrent chemotherapy. Other symptoms included dysuria (10%) and loose stools (16.7%).

Late toxicities primarily involved grade 2 lymphedema in 60% of patients, managed with physiotherapy and scrotal support. Meatal stenosis occurred in 16.7%, requiring dilation or meatotomy in some cases. Scrotal induration and periurethral fibrosis were noted in 16.7% and 6.7% of patients, respectively. One patient developed cutaneous angiosarcoma 6 years post RT. One patient was diagnosed with second primary lung adenocarcinoma 2 months post RT.

## Discussion

Penile cancer remains a rare and challenging malignancy, particularly concerning its treatment and management. This retrospective cohort study offers insights into the efficacy and safety of adjuvant RT in 30 patients treated over 14 years.

HPV status has emerged as an important factor influencing the response to RT in penile squamous cell carcinoma. At our center, routine HPV testing has been implemented since 2018. Multiple studies have consistently reported that HPV-positive tumors exhibit enhanced radiosensitivity, resulting in better response to RT in HPV-positive patients.^9^ In this context, Bandini et al^10^ analyzed data from a large multicenter cohort of patients with penile cancer who underwent ILND, with or without adjuvant therapy. Their analysis demonstrated that HPV-positive tumors were associated with significantly better survival outcomes when treated with RT, whereas a similar benefit was not seen in HPV-negative tumors. These findings highlight the prognostic and potentially predictive value of HPV status in guiding adjuvant treatment decisions for patients with penile cancer.

Although patients with higher T stage (particularly T4) exhibited a trend toward increased risk, the association was not statistically significant. The wide confidence intervals likely reflect the small sample size and limited number of events, which may have reduced the power to detect differences between subgroups. Nonetheless, the observed pattern aligns with existing literature indicating that increasing T stage generally correlates with adverse outcomes in penile cancer.^11^

All patients underwent bilateral ILND, while PLND was conducted in 90% of cases, with criteria evolving over the study period.^12^ Historically, PLND was reserved for cases with extensive inguinal nodal involvement or ENE. The high proportion of patients receiving bilateral PLND highlights the advanced disease stage in this cohort.

Among 14 patients staged as cN3, 13 demonstrated radiologic suspicion of pelvic nodal involvement, and 1 had suspected ENE. All 14 patients, along with an additional 13 patients (a total of 27 of 30), underwent PLND. Of the 13 patients with radiologically suspected pelvic nodal disease, only 4 had pathologically confirmed pelvic metastasis, while 9 were false positives. Furthermore, although only 1 patient had radiologic suspicion of ENE preoperatively, ENE was confirmed pathologically in 23 patients (76.7%). These findings underscore the limited sensitivity and specificity of conventional imaging for detecting pelvic nodal metastasis and ENE in penile cancer. Combined PET-CT, MRI and ultrasound-guided FNAC (Fine Needle Aspiration Cytology) may help refine pretreatment staging, particularly in high-risk disease, but further prospective evaluation is warranted to define their optimal role.

Adjuvant RT was delivered to all patients, irrespective of nodal burden, with doses ranging from 30.6 Gy to 65 Gy (median 50.4 Gy). While intensity modulated RT was the predominant RT modality (80%), earlier cases were treated with 2D or 3DCRT techniques (Table 1). Among patients treated with older modalities, recurrence rates were higher, underscoring advancements in radiation delivery methods. Ager et al's^13^ findings on the importance of delivering doses above 50 Gy in pN3 disease were validated in this study, emphasizing the role of appropriate dosing in improving local control.

Concurrent chemotherapy was administered to 26.6% of patients, primarily cisplatin-based regimens, and 3.3% had received NACT. Emerging evidence supports the role of NACT in patients with clinical N3 disease or nodes measuring 4 cm or greater, suggesting a paradigm shift toward combined-modality therapy in these cases.^14^

The median OS of 77.2 months in this study compares favorably with prior research. While Garg et al^15^ reported a median OS of 85 months in a cohort with 52.9% node-positive patients, the present study’s cohort included 93.3% node-positive patients, reflecting a higher-risk population and making the observed OS significant despite the more advanced disease profile.

The findings of our study should be interpreted in light of the small sample size, which likely contributed to some variables demonstrating trend-level associations without reaching statistical significance. Nonetheless, despite the limited cohort size, the trends observed in our analysis are consistent with previously published data and offer meaningful clinical insights. These findings add to the growing body of evidence supporting the role of adjuvant RT and multimodal therapy in node-positive disease, while also highlighting the need for collaborative, multicenter efforts to validate these results in larger populations.

Recurrence patterns revealed locoregional relapses as the predominant mode of failure. These findings align with prior studies by Leijte et al,^16^ which reported similar recurrence distributions. Leijte et al^16^ evaluated the pattern of recurrence in penile cancer, noting a recurrence rate of 29.3%, consisting of 18.6% local, 9.3% regional, and 1.4% distant recurrence. By comparison, our study showed a recurrence rate of 26.6%, comprising 20% locoregional recurrence and 6.6% distant metastasis. A recurrence rate of 33.3% (2/6 locoregional recurrences) was identified in the prepubic fat region, highlighting its clinical significance and the need for its inclusion in RT planning. This is observed in clinical audit after which prepubic fat was included after 2023. This observation is further supported by a study, as defined by the International Penile Advanced Cancer Trial, done by Cooper et al,^17^ which concluded that incorporation of the prepubic fat into the radiation field is essential.

Adjuvant RT demonstrated a clear benefit in reducing regional failures, as evidenced by Chen et al,^18^ who noted regional failure rates of 11% with RT versus 60% without RT. However, the presence of ENE remained a significant challenge, limiting the efficacy of RT as a standalone therapy. The findings of Frank et al^19^ suggest that adjuvant RT may not significantly improve outcomes for patients with ENE, contrasting with those without ENE. Studies by Johnstone et al^20^ and Tang et al^21^ underscore the limited efficacy of RT in ENE-positive cases. In Tang et al's^21^ study, although primarily focusing on high-risk groups, it does not explicitly clarify whether ENE-positive cases constituted the majority.^21^ However, their findings highlight the need for multimodal approaches, with adjuvant pelvic radiation showing an improved median time to recurrence of 7.7 months compared to 5.3 months for patients not receiving radiation. The inclusion of predominantly poor-prognosis cases likely explains the overall low OS of 12.2 months for radiated patients versus 8 months for nonradiated patients. This emphasizes the necessity of integrating chemotherapy in ENE-positive cases to improve survival outcomes.

However, more recent data suggest that the prognostic impact of ENE may be less absolute than previously thought. In our cohort, despite 76.7% of patients demonstrating ENE, survival outcomes were relatively favorable, indicating that RT may help mitigate its adverse effect when delivered within a structured treatment protocol. Ager et al^13^ similarly reported encouraging outcomes in a multicenter cohort comprising 98.6% of patients with ENE, achieving 5-year cancer-specific survival (CSS) and OS rates of 51% and 44%, respectively. The authors attributed these results to the use of standardized RT protocols and centralized management, reinforcing the role of adjuvant RT even in patients with extensive nodal disease (pN3). Together, these findings underscore that while ENE remains a marker of advanced disease, its prognostic value may vary across studies, likely due to differences in treatment strategies, radiation techniques, and patient selection. Further multicenter prospective studies are warranted to clarify the true impact of ENE and to identify which subgroups derive the most benefit from adjuvant therapy.

A meta-analysis by Zhou et al^22^ showed that patients with PNI had worse CSS and a higher cancer-specific mortality compared to those without PNI. However, there was no significant difference in OS between patients with and without PNI. Similarly, in a study by Li et al,^23^ it was demonstrated that patients with the presence of LVI had significantly lower OS. Nevertheless, our study did not reveal any correlation between PNI or LVI and OS or RFS.

In our cohort, the median primary tumor size was 4.0 cm (IQR, 2.45-4.50 cm), and increasing tumor size showed a strong adverse prognostic association. On univariate analysis, larger tumor size demonstrated a trend toward inferior RFS (HR 1.297, 95% CI, 0.979-1.717; P = .070) and OS (HR 1.290, 95% CI, 0.997-1.66; P = .053), suggesting that even incremental increases in tumor burden may confer worse outcomes. These findings are directionally consistent with the large SEER (Surveillance, Epidemiology, and End Results)-based analysis by Li et al,^24^ who reported that tumors ≥3 cm were associated with adverse pathologic features and significantly inferior OS and CSS, and remained an independent predictor on multivariable analysis (OS HR 1.665; P < .001). Although our results narrowly missed conventional thresholds for statistical significance, likely reflecting the limited sample size inherent to single-institution penile cancer series, the effect estimates were comparable to those in the SEER cohort and highlight the biologic relevance of tumor size as a prognostic factor. Taken together, these findings reinforce that primary tumor size may have meaningful clinical implications in penile cancer and support its consideration during risk stratification and treatment planning.

Toxicity remains a critical consideration, with grade 2 lymphedema observed in 60% of patients. Its severity tended to decrease over time. Higher grades of lymphedema were more frequently seen in patients treated with conventional or 3DCRT techniques, and in those who received boost RT for ENE-positive nodes. Secondary malignancies, though rare, underscore the importance of long-term follow-up. One patient developed postirradiation cutaneous angiosarcoma in the irradiated field, reflecting the risks associated with RT.

One patient developed a second primary lung adenocarcinoma 2 months after RT, confirmed histologically and treated appropriately. As this did not represent recurrence of penile cancer, he remained in the risk set for both RFS and OS analyses. He did not experience penile cancer recurrence during follow-up.

The role of adjuvant RT in penile cancer remains incompletely defined due to the rarity of the disease and limited prospective data. Ongoing trials like International Penile Advanced Cancer Trial aim to refine treatment paradigms, addressing key questions on sequencing and integration of surgery, chemotherapy, and RT.^25^

This study has several limitations. The retrospective design inherently introduces biases, including selection and recall bias, which may influence the observed outcomes. Furthermore, the study relied on institutional heterogeneous practice, which may not reflect broader treatment approaches or resource availability in other settings. The lack of a control group and prospective data further restricts the ability to draw definitive conclusions about the relative efficacy of adjuvant RT. In addition, the inability to perform multivariate analysis represents another limitation of this study. The small event number precluded reliable multivariable modeling despite exploratory penalized approaches. Lastly, missing data on certain patient characteristics and long-term toxicities may underrepresent the true extent of treatment-related effects.

The interpretation of our findings must take into account the limited sample size, which is an inherent challenge in studying rare malignancies such as penile cancer. Although our cohort is comparable in size to other contemporary series, the small number of events likely contributed to several associations demonstrating directional trends without reaching statistical significance. This limitation underscores an important reality in penile cancer research—single-center studies, even from high-volume institutions, are often underpowered to detect modest effect sizes. As a result, potentially clinically meaningful prognostic signals may remain statistically inconclusive. These findings highlight the need for multi-institutional collaboration and pooled prospective data sets to strengthen statistical power, validate emerging prognostic markers, and better define the role of adjuvant RT in high-risk subgroups.

## Conclusion

Although adjuvant RT was used, outcomes remain suboptimal with persistent locoregional failures. These results emphasize the need for enhanced multidisciplinary strategies and further research.

## Disclosures

None.
